# Using stable distributions to characterize proton pencil beams

**DOI:** 10.1002/mp.12876

**Published:** 2018-04-15

**Authors:** Frank Van den Heuvel, Ben George, Niek Schreuder, Francesca Fiorini

**Affiliations:** ^1^ CRUK/MRC Oxford Institute for Radiation Oncology University of Oxford Oxford UK; ^2^ Dept of Haematology/Oncology Oxford University Hospitals NHS Foundation Trust Oxford UK; ^3^ Department of Medical Physics Provision Center for Proton Therapy Knoxville TN USA

**Keywords:** dosimetry, lateral scatter, modeling, proton, protons

## Abstract

**Purpose:**

To introduce and evaluate the use of stable distributions as a methodology to quantify the behavior of proton pencil beams in a medium.

**Methods:**

The proton pencil beams of a clinically commissioned proton treatment facility are replicated in a Monte Carlo simulation system (FLUKA). For each available energy, the beam deposition in water medium is characterized by the dose deposition. Using a stable distribution methodology, each beam with a nominal energy *E* is characterized by the lateral spread at depth *z*:* S*(*z*;* α*,* γ*,* E*) and a total energy deposition *I*_*D*_(*z*,* E*). The parameter *α* describes the tailedness of the distributions, while *γ* is used to scale the size of the function. The beams can then be described completely by a function of the variation of the parameters with depth.

**Results:**

Quantitatively, the fit of the stable distributions, compared to those implemented in some standard treatment planning systems, are equivalent for all but the highest energies (i.e., 230 MeV/u). The decrease in goodness of fit makes this methodology comparable to a double Gaussian approach. The introduction of restricted linear combinations of stable distributions also resolves that particular case. More importantly, the meta‐parameterization (i.e., the description of the dose deposition by only providing the fitted parameters) allows for interpolation of nonmeasured data. In the case of the clinical commissioning data used in this paper, it was possible to only commission one out of five nominal energies to obtain a viable dataset, valid for all energies. An additional parameter *β* allows to describe asymmetric beam profiles as well.

**Conclusions:**

Stable distributions are intrinsically suited to describe proton pencil beams in a medium and provide a tool to quantify the propagation of proton beams in a medium.

## 1 Introduction

In proton beam therapy treatment planning, analytical descriptions of the treatment beams are commonly used to determine the dose deposited in clinical patient models. Although Monte Carlo‐based methods have become faster during the last few years, there is still a distinct advantage for using more efficient analytical models. In particular, if multiple calculations need to be performed, such as in the process of four‐dimensional (4D)‐robust‐optimization or adaptive therapy. This advantage is significant in the case of pencil beam‐based proton therapy, where multiple small beams need to be tracked and calculated. However, the currently implemented analytical algorithms have been shown to be less reliable in more complex clinical treatment sites like lung and breast,^1^, ^2^ which necessitate a more accurate description of the dose deposited by a scanned proton beam. Yet, an improved analytical description of the lateral pencil beam model can provide a better representation of the macroscopic processes of a pencil beam in a medium, but still preserving the calculation speed characteristic of a TPS, nonstochastic, algorithm.

A pencil beam entering a medium will generate secondary particles, such as scattered neutrons, photons, *δ*‐rays, and large angle scattered protons, producing a *nuclear halo* of dose around the central beam axis. The relative contributions of scattered protons, *δ*‐rays, and neutrons change as the beam penetrates deeper in the medium. Although the contribution from a single pencil beam is small in a region far away from the central axis, a complex treatment plan is made of many pencil beams and the summation of the lateral contributions could be significant.

The description and treatment of the nuclear halo has been the focus of research by a number of groups who have proposed various methodologies describing the effects in an analytical way. Gottschalk et al. presents an in‐depth analysis of all the physical processes contributing to the beam lateral profile, subdividing a pencil beam into a combination of four distinct regions: core, halo, aura, and (possibly) spray.^3^, ^4^ Such a detailed approach requires up to 25 different physical parameters to characterize the beam, making it logistically difficult to implement. Therefore, in most commercially available implementations, the contribution from the nuclear halo is quantified by adding different distributions to a central Gaussian distribution describing the core of the pencil beam.

In a first instance, the lateral scatter behavior as a function of the radial distance *r* of proton pencil beams, of energy *E*, in air are described by a radially symmetric two‐dimensional (2D) normal distribution:

(1) $$S\left(r;E\right)=\frac{1}{2\pi \sigma^{2}\left(E\right)}\text{exp}\left(-\frac{r^{2}}{2\sigma^{2}\left(E\right)}\right)$$

with *σ* the variance of the normal distribution. Once a beam propagates in a solid medium, the halo component becomes more important. A first proposed solution for the nuclear halo by Pedroni et al. added another, broader Gaussian to the core; a methodology which is implemented in the Varian Eclipse (Varian Medical Systems, Palo Alto, CA) calculation algorithm.^5^, ^6^ This can be parameterized as follows:

(2) $$\begin{array}{cc} S(r;z,E)= & \frac{1-q}{2\pi \sigma_{1}^{2}\left(z,E\right)}\text{exp}\left(-\frac{r^{2}}{2\sigma_{1}^{2}\left(z,E\right)}\right)\\ & +\frac{\left(q\right)}{2\pi \sigma_{2}^{2}\left(z,E\right)}\text{exp}\left(-\frac{r^{2}}{2\sigma_{2}^{2}\left(z,E\right)}\right) \end{array}$$

where *z* represents the depth in a medium. The same notation was followed as in Eq. (1), *σ*_1_ and *σ*_2_ denoting the different normal distributions. The parameter *q* provides the relative contribution of each Gaussian.

Another clinically used algorithm for pencil beam calculation is implemented in RayStation^TM^ TPS (RaySearch Laboratories, Stockholm, Sweden). In this system, the lateral dose is modelled as a superposition of 19 Gaussian distributions (19 subspots: 1 at the center, and 6 and 12 positioned at two concentric circles around the center).^7^

In further refinements of this approach, other groups attempted combinations of Gaussian, Lorentz (also known as Cauchy), and Lévy distribution functions,^8^ increasing the complexity of the fitting procedure and necessitating look‐up tables for the various parameters. It is interesting to note that a number of the more successful methods combine two or more stable functions in their analytical representation. However, when having so many parameters, one could question whether it would not be more advantageous and correct to use the approach proposed by Gottschalk (i.e., modeling the physical parameters) or alternatively, applying a spline fit to measured data, to describe the pencil beam.

In this paper, we review the concept of stable distributions and show that they can be used to represent the evolution of a proton pencil beam in a medium. Indeed, the notion of a stable distribution allows to describe combinations of different distributions. These uni‐modal distributions can not only have variable width but also different contributions in their tails, both of which can be quantified by the parameters *γ* and *α*, respectively. Moreover, the fact that stable distributions represent an infinity of distribution types, allows a description of how such a distribution, quantified by its parameters, changes over time, or in this case as a pencil beam penetrates deeper in a medium.

While there is no direct mechanistic basis to this description, the tailedness (*α*) of the distribution is mainly influenced by the halo, while the overall broadness (*γ*) is governed by the beam divergence combined with primary scatter processes.

We demonstrate that this approach provides an accurate description of the pencil beam when compared to published measured data and geometrically extended Monte Carlo simulations. In addition, we investigate the behavior at higher energies and show that a more complex methodology is needed. Finally, we show that the description of the pencil beam behavior as a function of depth in the medium can be investigated by a parametric approach, whereby the parameters are a well‐behaved function of penetration depth. This can lead to further research in quantifying these parameters in nonhomogeneous media as well as characterizing the medium traversed expressed as changes in these parameters.

## 2 Materials and methods

### 2.A. Stable distributions

Stable distributions are a class of distributions which generalize a property of the normal distribution. Namely, they extend the central limit theorem which states that if the number of samples drawn from random variables, *with or without* finite variance, tends to infinity, then the measured distribution tends to a stable distribution. If the variance is finite, the resultant distribution tends toward the normal distribution, a member of the class of stable distributions. A good introduction to this subject can be found in work by Nolan et al.^9^ A more extensive review is available by Uchaikin and Zolotarev.^10^

Other than for specific cases, these distributions do not possess an analytical representation. It is, therefore, necessary to describe them in terms of their characteristic function which always exists for a given stable distribution.

More generally, the characteristic function, *φ*(*t*), of a distribution is the Fourier transform of the probability density function, *f*(*x*), of that distribution, for example,

(3) $$\varphi \left(t\right)=\frac{1}{2\pi }\int_{-\infty }^{\infty }f\left(x\right)e^{-ixt}dx$$

It can be shown that all stable distributions have an identical characteristic function, *φ*(*t*), barring a change in the parameters (*α*,* β*,* γ*,* δ*):

(4) $$\varphi \left(t;\alpha ,\;\beta ,\;\gamma ,\;\delta \right)=\text{exp}[it\delta -|\gamma t{|}^{\alpha }\left(1-i\beta \text{sgn}\left(t\right)\phi \left(t\right)\right)]$$

with *ϕ*(*t*) = tan (*πα*/2) except for *α* = 1, in which case $\phi \left(t\right)\;=\;-\frac{2}{\pi }\text{log}\left(t\right)$. The parameter *α* ∈ [0, 2] determines the shape of the distribution, *β* ∈ [−1, 1] is a measure for symmetry, *γ* ∈ [0, +∞] is a scale factor and *δ* a position, or the most probable value.^10^ For a symmetric, zero‐centered distribution, the Eq. (4) reduces to:

(5) $$\varphi \left(t;\alpha ,\gamma \right)=\text{exp}(-|\gamma t{|}^{\alpha })$$

In the supporting material of this paper we show from first principles that this equation represents all symmetric zero‐centered distributions that follow the central limit theorem.

As *α* and *γ* can vary continuously there are an infinite number of stable distributions, most of which do not have an analytical representation in real space. Indeed, only for *α* = 2, 1, and 0.5 (*β* = 1) a closed analytical form is known. These correspond to the Gauss, Lorenz, and Lévy distributions, respectively.

Using this generalization it is possible to define a class of unimodal distributions whose properties can be exploited to describe physical random walk processes which combine different physical properties.^11^

In the remainder of the paper, we will denote a stable distribution related to a pencil beam of energy *E* with a variable *r* and the parameters *α*,* β*,* γ*, and *δ* by

(6) S(r;z,E,α(z;E),β(z;E),γ(z;E),δ(z;E))

The parameters depend on the depth *z* along the central axis, which is denoted by writing the parameters as a function of *z*. In the special case of a symmetric zero‐centered distribution (i.e., *β* = *δ* = 0) we denote **S**(*r*;* z*,* E*,* α*(*z*;* E*), *γ*(*z*;* E*)). For brevity and readability we will forego the explicit mention of the depth *z* and beam energy *E*, in the parameters using **S**(*r*;* z*,* E*,* α*,* γ*).

### 2.B. Measured data

To test the stable distribution approach we used data presented in an article by Bellinzona and colleagues,^8^ attached to the CNAO (Centro Nazionale di Adroterapia Oncologica) Pavia, Italy. They graciously provided the raw data from their experiments. Measured data are available from a synchrotron‐based proton facility down to a level of 5 × 10^−3^ of the maximal dose, where the measurement error was estimated to be of the order of 0.5%. The measured data were acquired using a pin‐point chamber from 2.5 to 4.5 cm distance from the central axis (CAX), depending on the energy of the proton beam. The data are normalized to the CAX measurement. In the article, they also show good to reasonable agreement (*χ* ^2^/NDF ≃ 1−3) with FLUKA‐based Monte Carlo simulations, albeit still for a limited distance from the CAX (< 5 cm). The data in the article are for proton beams of energy 117.75 and 154.4 MeV only. Two phantom distances were investigated: for the lowest energy at 2.5 cm and 8 cm depth, and for the highest energy at 10 and 15.2 cm. As a comparison these data are presented here. The data provided by the Bellinzona group were more extensive than the one presented in their publication, and included data from proton pencil beams with energies up to 174 MeV. All data available from CNAO have been fit using stable distributions. These more extensive results are tabulated in the supplementary data section. In the main part of this article, we only report on the energies and distances available in the article published by Bellinzona et al.

### 2.C. Monte Carlo simulations

The data presented by CNAO are limited in lateral range and are reliable only up to three orders of magnitude from the maximum and no more than 4.5 cm off‐axis. Data on beam energies higher than 174 MeV were not available. We expanded the simulation dataset in lateral range and available energies. This is to better evaluate the parameterization. The Monte Carlo‐simulated data were generated for another proton center: the ProVision Center for Proton Therapy in Knoxville, Tennessee, US. This center is currently operational using an IBA cyclotron which provides proton beam scanning technique up to a maximum energy of 230 MeV. The device can generate beam energies at given discrete energy levels, which we label as the nominal energy. The latter is defined as the energy of a mono‐energetic beam having the same range as the position of the 90% beam maximum (at the Bragg peak) of the physical beam in water. For this work, the ProVision beams from 98 to 230 MeV were reproduced with the Monte Carlo code FLUKA,^12^, ^13^ by adapting the simulated beams to the commissioning experimental beam data.^14^ At each energy, the beam is defined at the surface of the phantom by a 2D normal distribution characterized by position and standard deviation, *σ*. Using FLUKA, the dose distribution in medium (water) is calculated in a 200 × 200 × 350 mm^3^ cube with 1 mm^3^ voxels. The calculated dose distribution in each 200 × 200 slice perpendicular to the beam axis is then considered to be a two‐dimensional dose distribution. Generation of secondary particles under the form of high‐energy photons (i.e., photons energetic enough to produce ionization), neutrons, and *δ*‐rays was enabled explicitly, while the contribution of large angled scattered protons is automatically tallied.

To evaluate the parameterization, three energies (low, middle, and high) were chosen and the lateral dose deposition was calculated up to 10 cm from the CAX. Both the highest energy and the CAX distance are larger compared to the data obtained from Bellinzona and colleagues.

### 2.D. Fitting procedures

Because the majority of stable distributions do not possess an analytic form, it is difficult to use the classical approach to fit the data to a curve representing a pencil beam. Indeed, the fitting of stable distributions is the subject of scientific research by itself. We opted to use a maximum likelihood estimation based on precomputed spline approximations.^9^ In essence, it selects the parameters of precomputed stable distributions to best match the distributions which generates the same probability density function as the calculated lateral scatter.

Once the parameters are determined, the characteristic function is calculated in complex space and using a numerically calculated inverse Fourier transform the actual stable distribution was generated. A straightforward methodology also proposed by Mittnik et al.^15^

Calculation of alpha‐stable distributions is performed using a fast, parallel, high‐precision C/C++ library, *libstable*.^16^

### 2.E. Goodness of fit

Once the parameterization is known at every given depth, it is necessary to provide an estimate of the goodness of fit of the model to the measured data. Classically, this is done using a reduced *χ* ^2^‐analysis, where *χ* ^2^ is defined by Eq. (7).

(7) $$\chi^{2}=\sum_{i=1}^{N}{\left(\frac{S_{i}-S_{i}}{\sigma_{i}}\right)}^{2}$$

where *S*_*i*_ is the reference value determined with an associated error estimate *σ*_*i*_. This is compared to the expressions derived by the model **S**_*i*_. The reduced *χ* ^2^ is obtained by dividing the resulting sum by the degrees of freedom of the whole system: that is, the number of measured points minus the number of parameters in the model. As a rule of thumb a reduced *χ* ^2^‐value of 1 indicates a good fit as overall the value of the error is comparable to the measurement error. A higher value indicates that the model does not represent the data well and a lower value could be an indication that the model overfits the data, or that the error estimate is too large.

WhilE the reduced‐*χ* ^2^ approach is standard practice it does not provide good results in cases where the model under consideration is highly nonlinear, as is the case here. By highly nonlinear we mean that there are parts within the function that differ by orders of magnitude. To be specific, a major factor in this case lies in the quantification of the tail of the distribution making a standard reduced *χ* ^2^‐analysis unsuitable. To provide a more valid analysis, it is necessary to analyze the log _10_ transformation of the data. Under these conditions, the error estimate for the transformed measured data needs to be used. This can be found through error propagation:

(8) $$\begin{array}{cc} \sigma_{{\text{log}}_{10}\left(X\right)} & =\sigma_{X}\frac{\partial {\text{log}}_{10}\left(X\right)}{\partial x}\\ & =\sigma_{X}\frac{1}{\text{ln}(10)}\frac{\partial \text{ln}(X)}{\partial x}=0.434\frac{\sigma_{X}}{X} \end{array}$$

It is now possible to calculate the *χ* ^2^ value as follows:

(9) $$\begin{array}{c} \chi^{2}=\sum_{i=1}^{N}{\left(\frac{{\text{log}}_{10}\left(S_{i}\right)-{\text{log}}_{10}\left(S_{i}\right)}{0.434\sigma_{i}/S_{i}}\right)}^{2} \end{array}$$

It is this expression [Eq. (9)] which was used to calculate the goodness of fit for the measured data. This methodology is suited to calculate the goodness of fit for the measured data provided by CNAO, as the measurement errors are known. An added complication in the case of Monte Carlo simulations is that the error on the simulated measurement data change with the amount of energy deposited. This results in very small errors in high‐dose regions and very large error estimates in low‐dose regions. In the *χ* ^2^ expression, the difference between measured and predicted value is weighted by the inverse of the error, making the value of *χ* ^2^ dominated by the peak values rather than the tails which are of importance here. To deal with this and provide meaningful *χ* ^2^‐values, we introduce a minimal error in the Monte Carlo simulation data. The exact minimal error used is not critical for reasonable estimates of the error (data not shown) and we chose the value to be the same as the error value provided in the CNAO data (i.e., 5%). While this methodology cannot be used to obtain an absolute value of the goodness of fit, it allows relative assessment of the model to the measured data.^17^

Using this approach, the goodness of fit of the stable distribution model is calculated at all depths for three nominal energies (100.32, 176.18, and 226.08 MeV).

### 2.F. Reference data

Purely as a comparative tool we generated double Gaussian fits to our data in some of the cases. This uses a standard Levenberg–Marquardt minimization^18^, ^19^ of Expression (2).

### 2.G. Parameterization and scaling

For all pencil beams, as commissioned in the Knoxville treatment facility, with nominal energy (*E*_*n*_), we extracted the centrally located transverse distribution at all available depths (*z*). From now on, by denoting the radial variable of this extraction as *r* instead of *x* or *y*, we imply that the beam is considered to be radially symmetric. Calculating the dose and normalizing this in a plane perpendicular to the beam direction, yields the laterally scattered dose *S*(*r*;* E*_*n*_, *z*). Subsequently, the normalized stable distribution parameters *α*(*z*;* E*_*n*_) and *γ*(*z*;* E*_*n*_) are determined using the above‐mentioned fitting procedure. This distribution is denoted by **S**(*r*;* z*,* E*_*n*_, *α*,* γ*). In a first approximation we consider the pencil beams to be radially symmetric. Finally, the total integral dose at each depth *I*_*D*_(*z*;* E*_*n*_) is also calculated. This procedure yields three parameters which vary as a function of depth and nominal beam energy allowing us to calculate the dose distribution at any depth in a homogeneous medium.

Finally, to reconstruct the dose deposition of a given energy in a homogeneous medium it suffices to multiply the distribution with the total dose deposited in that plane, which yields a reconstruction of the dose delivered.

(10) $$\begin{array}{cc} D(r;\;z,\;E_{n},\;\alpha \left(z\right),\;\gamma \left(z\right))=\;\; & I_{D}\left(z,\;E_{n}\right)S(r;\;z,\;E_{n},\;\\ & \times \alpha \left(z;\;E_{n}\right),\gamma \left(z;\;E_{n}\right)) \end{array}$$

### 2.H. Data interpolation

We propose a methodology to determine the beam characteristics of intermediate energies from two provided beam characterizations.

Conjecture 1 *(Intermediate Morphing)*. Let *α*(*z*,*E*_*i*_), *γ*(*z*,*E*_*i*_), and *D*(*z*,*E*_*i*_) be the parameters fully describing a proton pencil beam with nominal energy E_i_. Then it is possible to calculate the parameterization of an intermediate energy E_i_ by interpolation of the parameterization of energies E_l_ and E_u_, which are respectively lower and higher compared to E_i_. This is irrespective of a scaling in the depth parameter which depends on the range of the given energy.

Let E_l_ < E_i_ < E_u_ :

(11) $$\alpha \left(z_{i};E_{i}\right)=\alpha \left(z_{l};E_{l}\right)+\frac{E_{i}-E_{l}}{E_{u}-E_{l}}\left(\alpha \left(z_{u};E_{u}\right)-\alpha \left(z_{l};E_{l}\right)\right)$$

(12) $$\gamma \left(z_{i};E_{i}\right)=\gamma \left(z_{l};E_{l}\right)+\frac{E_{i}-E_{l}}{E_{u}-E_{l}}\left(\gamma \left(z_{u};E_{u}\right)-\gamma \left(z_{l};E_{l}\right)\right)$$

(13) $$D\left(z_{i};E_{i}\right)=D\left(z_{l};E_{l}\right)+\frac{E_{i}-E_{l}}{E_{u}-E_{l}}\left(D\left(z_{u};E_{u}\right)-D\left(z_{l};E_{l}\right)\right)$$

where: z_i_ = z_l_ℜ(E_i_)/ℜ(E_l_), with ℜ(E) being the range of a proton in the medium under consideration.

Using the methodology of intermediate morphing, we determine the minimal amount of beams we need to fully characterize in order to obtain a full set of data across all nominal energies. We chose a threshold of 1% error determining the width of the beam (*γ*) and 3% for the shape (tailedness) (*α*). The total deposited energy needs to be correct to 1% dose and 1 mm position.

## 3 Results

### 3.A. Measured data

**Figure 1:**
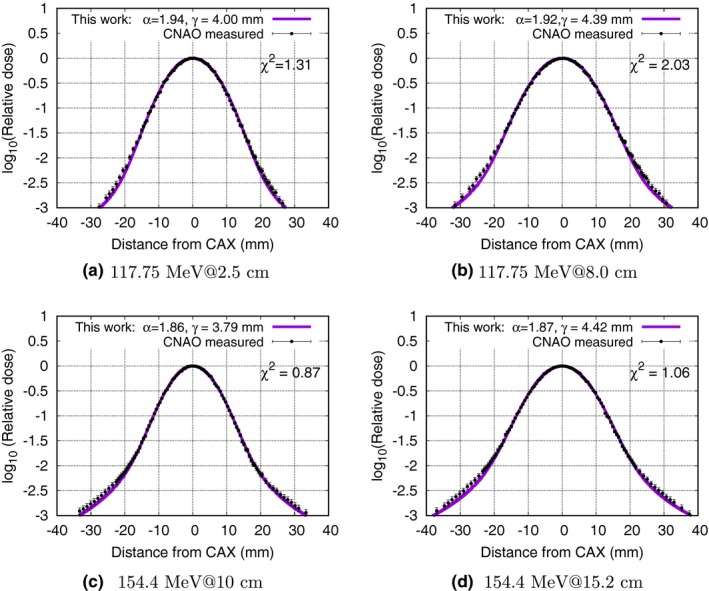
Lateral dose deposition for energies 117.75 and 154.4 MeV, recreated from measurements at CNAO (Bellinzona et al.^8^ (circles) and compared to stable distributions (solid line)). [Color figure can be viewed at wileyonlinelibrary.com]

In Fig. 1 (a), a selection of relevant stable distribution fits are displayed for the same energies and depths as provided in the article by the Bellinzona group:^8^ 117.75 MeV at 2.5 cm and 8 cm depth, and 154.4 MeV at 10 cm and 15.2 cm depth. Visual inspection of the curves show excellent agreement and all reduced *χ* ^2^ values are close to 1. With *χ* ^2^ calculated according to Eq. (7) as explained above. We repeated this effort using the full measurement dataset from CNAO. The result of which can be found in the supplementary dataset.

### 3.B. Simulations

**Figure 2:**
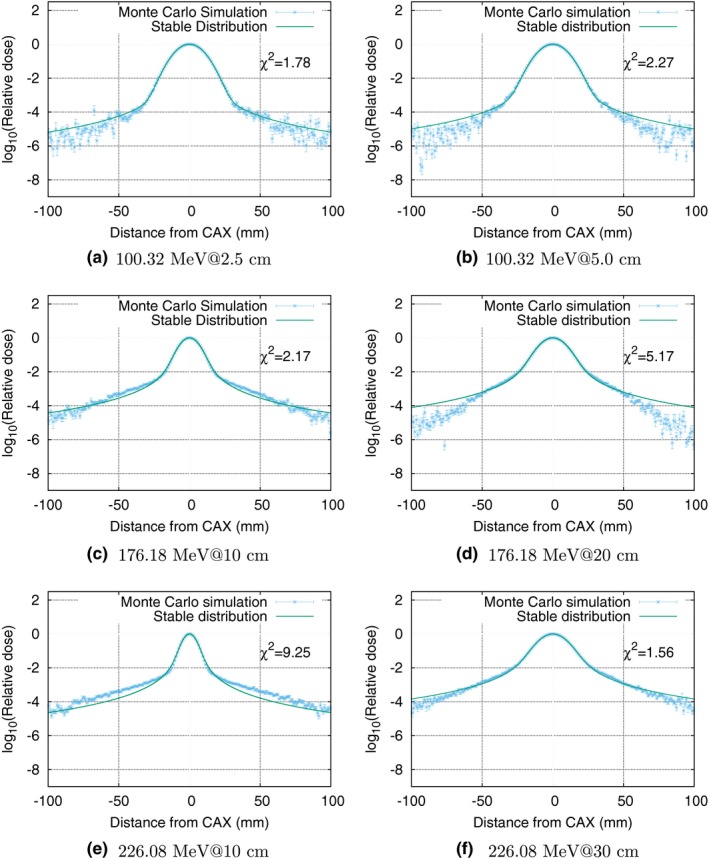
Lateral dose deposition calculated using Monte Carlo techniques for three different energies (100.32, 176.18, and 226.08 MeV) at two respective relevant depths (2.5 cm and 5 cm, 10 cm and 20 cm, and 10 cm and 30 cm). The Monte Carlo calculations depicted in dots and the fitted stable distributions as solid lines. [Color figure can be viewed at wileyonlinelibrary.com]

Figure 2 shows lateral dose depositions for a 100.32 MeV pencil beam at 2.5 and 5 cm deep, a 176.16 MeV pencil beam at 10 cm and 20 cm deep, and a 226.08 MeV pencil beam at 10, 20, and 30 cm deep. It is clear that while the values for the lower energies provide excellent agreement, the higher energies are not optimal. Visual inspection of the curves in Fig. 2 indicates that the tails in the intermediate distances from the central axis are underestimated by the model.

**Figure 3:**
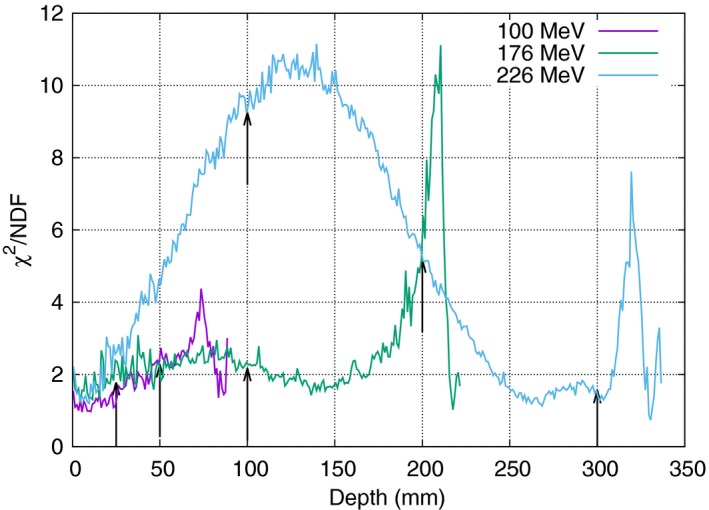
Quantifying the goodness of fit for stable distributions calculating the *χ* ^2^/NDF–value. Both lower energy curves exhibit good agreement, up until the Bragg peak, after which a small but systematic overestimation of the tail values is noted, as the distribution tends to a pure gaussian, due to the high contribution of primary proton dose deposition. The highest energy shows a region of underestimation of the tail values. While the differences still are very small an additional normally distributed background is able to correct for this. All *χ* ^2^/NDF–values in Fig. 2 are pointed out using arrows in this figure. Bragg peak positions: 79.5, 210.5, and 312.5 cm for the 100, 176, and 226 MeV curves, respectively. [Color figure can be viewed at wileyonlinelibrary.com]

As shown in Fig. 3, the goodness of fit deteriorates with the depth, the *χ* ^2^/NDF reaches maximum values close to the Bragg peak. However, this is not the case for the highest considered energy (226 MeV) where the *χ* ^2^/NDF is up to the order of 10 also for intermediate depths.

In a further effort to provide better agreement we can add a Gaussian background to the expression. Mathematically, the calculation does not become much more complex. However, some of the advantages of the stable distribution will be lost, as we start to over parameterize the problem. Two additional parameters are needed: firstly, a spread factor *σ* and secondly, a relative contribution, denoted *q*. Let **S**(*r*;* z*,* α*(*z*), *γ*_1_(*z*)) be the stable distribution at depth z. We define *N*(*r*;* z*,* σ*) to be the normally distributed background. The full expression now becomes:

(14) $$\left(1-q\right)S(r;\;z,\;\alpha \left(z\right),\;\gamma_{1}\left(z\right)+qN\left(r;\;z,\;\sigma \right)$$

**Figure 4:**
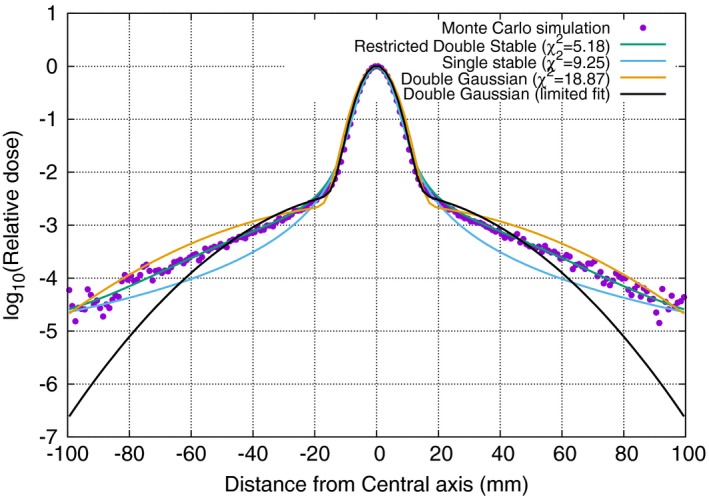
Dual stable distribution fit to 226 MeV beam profile at 10 cm deep. The model now correctly fits the contribution of large angle scattered protons. For comparison a single stable distribution (*χ* ^2^/NDF = 9.25 and a double Gaussian approach *χ* ^2^/NDF = 18.87 is also shown). [Color figure can be viewed at wileyonlinelibrary.com]

Taking into account that a normal distribution is a stable distribution with *α* = 2 and $\gamma_{2}\;=\;\sigma /\sqrt{2}$. It becomes clear that the characteristic function of the new expression is:

(15) $$\varphi \left(t\right)=\left(1-q\right)\text{exp}(-|\gamma_{1}{t|}^{\alpha })+q\text{exp}(-|\gamma_{2}t{|}^{2})$$

where *q* has the same role as in the double Gaussian expression. This new expression can be evaluated in the same manner as the regular stable distribution expression with a small computational penalty. Using an iterative approach, an optimal addition of an analytical normal distribution was found, using a Levenberg–Marquardt minimization procedure with relative contribution (*q*) and spread (*σ*) as parameters in the fitting procedure at each depth (*z*). Figure 4 shows the representation at 10 cm deep for a 226 MeV beam after an iterative procedure where the original stable distribution is used as a starting point. The normal distribution is then fit, after which the stable distribution is determined again based on the original minus the fitted normal distribution. Finally, the normal distribution is fit again. This results in a *χ* ^2^/NDF = 1.04 using the Levenberg–Marquardt fit for the Gaussian component alone and *χ* ^2^/NDF = 5.18 using Eq. (9), the latter is compared to a value of *χ* ^2^/NDF = 9.25 for the stable distribution alone and *χ* ^2^/NDF = 18.87 for a double Gaussian. Also of note is that in the latter case the values of the parameters depend heavily on the distance from the central axis taken into account during the fit process. The stable distribution approach, even without an added Gaussian shows almost no dependency on the width used to assess the lateral scatter. Table 1 provides the data to support this statement. Both the width parameters (*σ*_1_ and *σ*_2_) show high variability with the range. In practice this implies that the size of a detector (i.e., a film or a flat panel) used to measure the scatter will affect the values of a double Gaussian fit. This is much less the case when using the stable distribution formalism.

**Table 1:** The parameters estimated in the case of the double Gaussian (first two rows) are highly dependent on the range used to estimate the lateral scatter. In the case of stable distributions, the variation in the *γ* parameter is of the order of hundreds to thousands of a millimeter. The *α* parameter is not only slightly more susceptible to changes but also has a small impact on the shape of the curve

Parameter	10 cm	15 cm	20 cm
*σ*_1_	3.99 mm	4.17 mm	4.33 mm
*σ*_2_	22.46 mm	28.51 mm	32.18 mm
*α*	1.803	1.806	1.821
*γ*	2.663 mm	2.666 mm	2.673 mm

### 3.C. Parameterization

**Figure 5:**
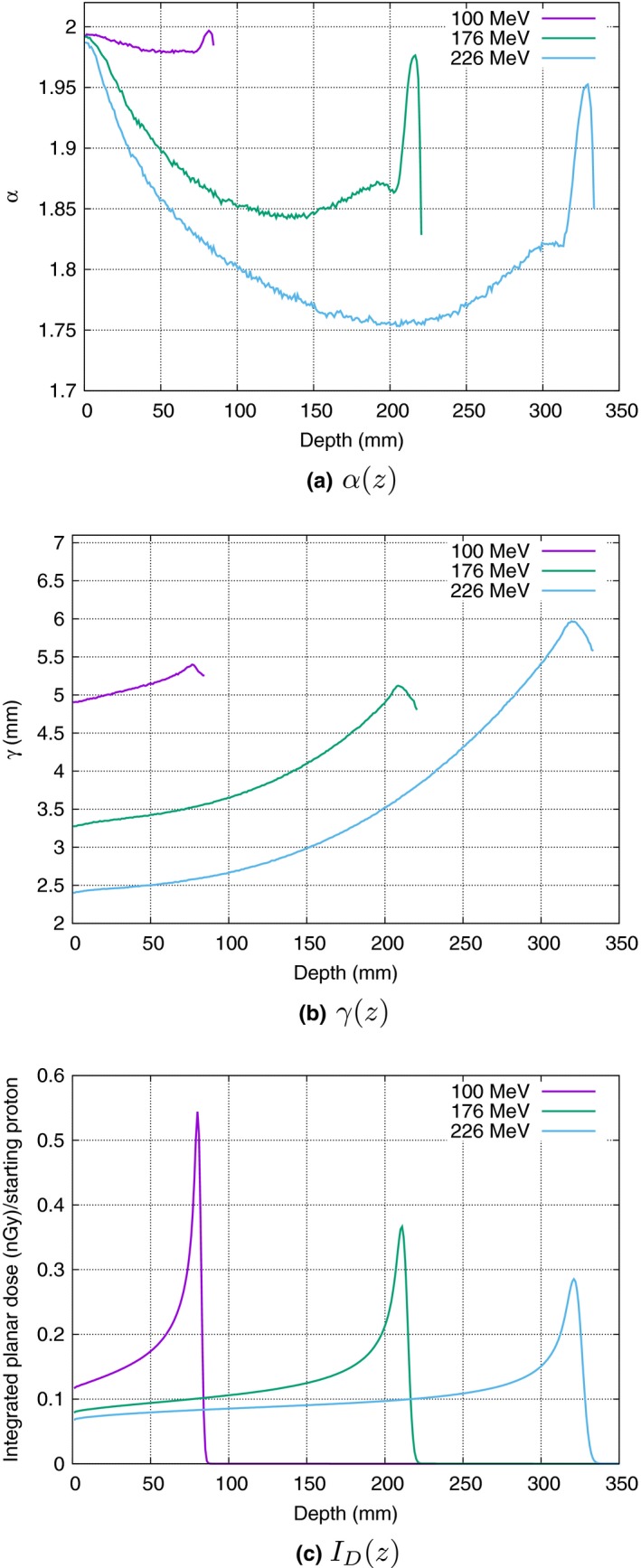
From top to bottom: the evolution of *α*,* γ*, and integrated dose, for the energies 100, 176, and 226 MeV. Note that the graph for each energy has the same general shape. A graph for all the energies in the Knoxville IBA machine can be found in the additional material. [Color figure can be viewed at wileyonlinelibrary.com]

The behavior of a proton pencil beam, as commissioned at the ProVision facility can be parameterized at a given depth and for a specific nominal energy using two parameters from the stable distribution fit: *α*, describing the tail of the distribution and *γ* providing the width. These parameters provide a normalized distribution. A final parameter is the integral dose *I*_*D*_ deposited at that depth [Fig. 5(c)]. The *α* parameter reflects the increasing contribution of interactions with longer range, most likely from scattered protons. The contribution diminishes due to two factors: (a) The decrease of generated secondary protons due to the lower energy of the primary protons, and (b) the decrease in energy of the secondary protons.

### 3.D. Interpolation of data

**Figure 6:**
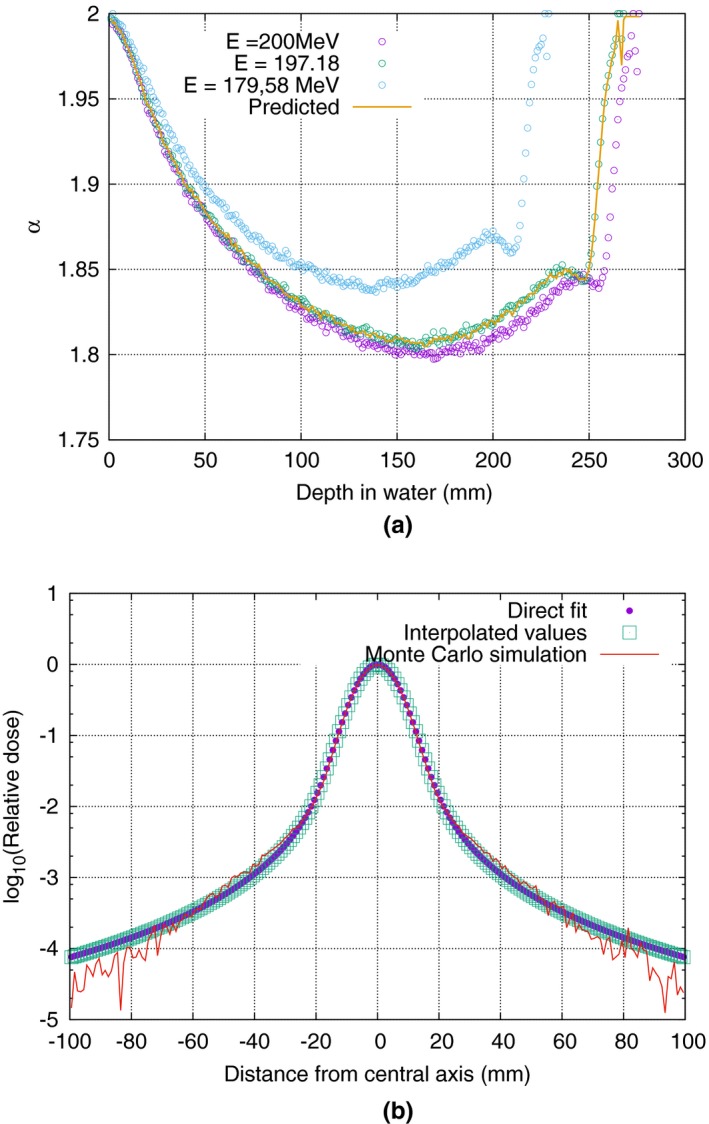
(a) Predicting the next *α* graph for a nominal energy of 197.18 MeV, using two outer source curves, with nominal energies 179.58 and 200 MeV. The line represents the *α* values for the interpolated curve, while the points represent the *α* values for the original curves. (b) Comparison between the lateral scatter as predicted by the stable distribution (lower circles) which was fit directly to the Monte Carlo‐simulated scatter (higher circles), with the stable distribution predicted by interpolation (middle circles). [Color figure can be viewed at wileyonlinelibrary.com]

Figure 6 shows the methodology interpolating the data from two energies to generate data for a third energy beamlet. We found the maximally allowed difference (i.e., *γ* < 1 mm, *α* < 3%) between interpolated and measured parametric representation. Due to the nonlinearity of the parameters’ behavior as a function of energy we expect that linear interpolation is useful only in a limited energy range. Indeed, it was shown that the parameter *γ* is the most sensitive and deviates to more than 1% if the interpolated energies are more than 20 MeV apart in nominal energy. The *α* parameter is not very sensitive to interpolating distance as the curve is relatively noisy.

Figure 6 illustrates the high level of agreement of the lateral scatter as calculated starting from a fitted stable distribution, compared to one obtained from interpolated *α*,* γ*, and *I*_*D*_ values. The comparison is almost perfect. The lateral scatter profile presented here is estimated at a depth of 200 mm. Plots at 50, 100, 150, and 240 mm, were also obtained with comparable results (data not shown).

### 3.E. Implementation in matRad

To allow testing of our parameterized beam model with clinical patient plans, we implemented the stable distribution dose calculation in an open source treatment planning system, matRad (DKFZ, Heidelberg, DE). matRad^20^, ^21^ is written in MATLAB (MathWorks, Natick, MA) and provides functionality for importing patient data, ray tracing, inverse planning, and treatment plan visualization. The proton dose calculation component was extended to support a beam model described by a stable distribution, in addition to the existing single and double Gaussian models.

Within matRad, for particle dose calculations, the dose to a voxel *d*_*i*_ from a pencil beam of energy *E* is the product of a depth‐dependent part and a lateral part. Firstly, the water‐equivalent path length, *z*, along the central beam axis to the depth of the voxel *d*_*i*_ is calculated. This value is used to interpolate tabulated depth dose data to determine the integral dose at depth *z*. Secondly, the off‐axis distance, *x*, to the point of interest is calculated. For the lateral beam broadening, matRad uses a set of depth‐dependent parameters for the required parameterized, (*σ*) for a single Gaussian or (*σ*_1_, *σ*_2_) for a double Gaussian. The lateral component is calculated as the value of the distribution at distance *x*. The distribution is normalized such that the integral over two dimensions is unity.

To implement our parameterization, we created tabulated depth dose curves from our FLUKA simulations to allow calculation of the integral dose at depth *z*. For the lateral broadening of the beam, we tabulated *α* and *γ* against depth for each proton energy. We extended matRad to include calculation of the stable distribution for beam broadening with the parameters.

To provide a radially symmetric beam, a 2D normalization is required when computing the lateral profile in a plane of distance *z* into a medium. As we assume radial symmetry, we denote this by using *r* as a variable in the stable distribution expression rather than *x*. If **S**(*r*;* z*,* α*,* γ*) is the value of the stable distribution that describes the 1D beam profile at a distance *r* from the central axis, the 2D beam profile is described by:

(16) $$L=\frac{1}{V}S\left(r;\;z,\;E_{n},\;\alpha \left(z;E_{n}\right),\gamma \left(z;E_{n}\right)\right)$$

where *r* is the distance from the pencil beam central axis and *α* and *γ* are the parameterization at depth *z*. *V* is the normalization required such that the volume under the 2D distribution is unity and is calculated using the shell formula as:

(17) $$V=2\pi \int_{0}^{\infty }rS\left(r;\;z,\;E_{n},\;\alpha ,\;\gamma \right)dr$$

Numerical computation of this integral is performed to provide the normalization. In our implementation we precalculated *V* for each combination of *α* and *γ* in the discrete beam parameterization and interpolated it as required within the dose calculation engine. *V* is shown to vary smoothly within the relevant range of parameter space.

**Figure 7:**
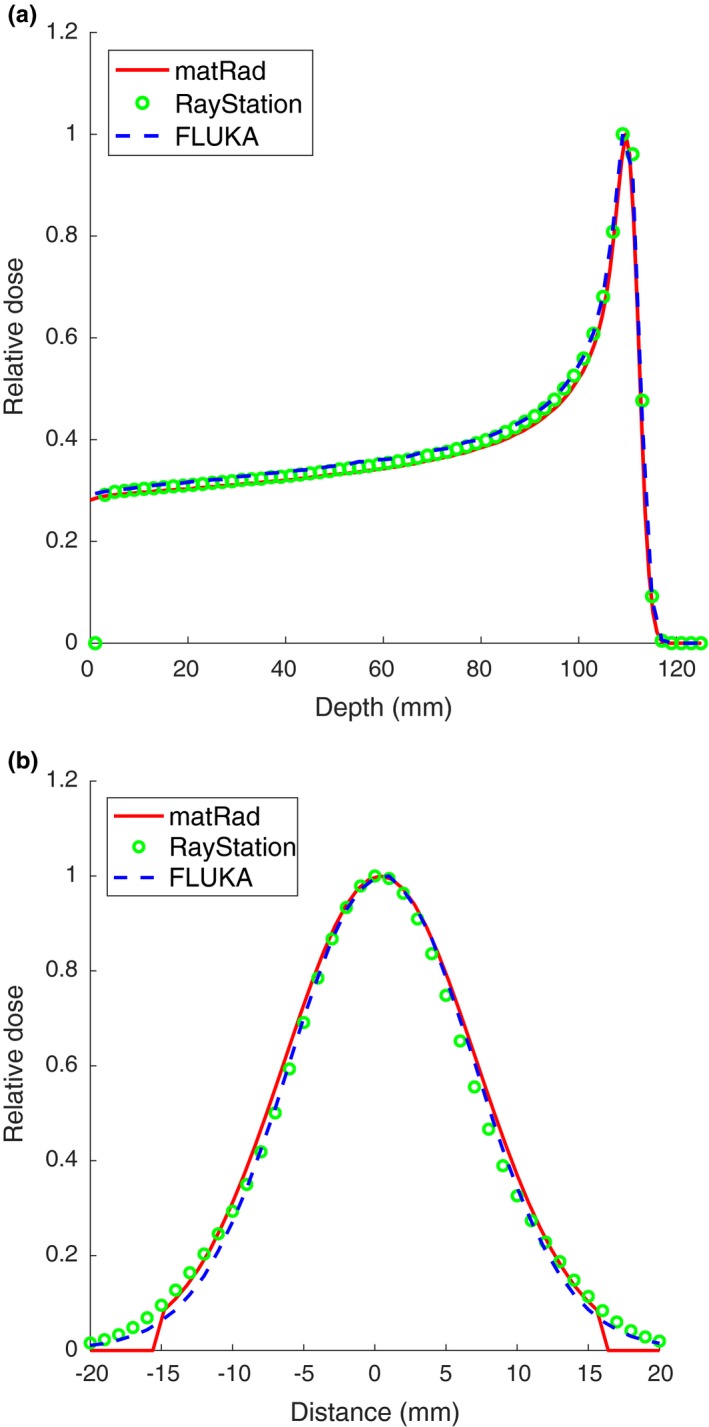
Comparison of matRad and RayStation calculations of a 120 MeV proton pencil beam dose distribution. The depth dose curve is calculated at the central axis position. The depth of the lateral beam is 50 mm. [Color figure can be viewed at wileyonlinelibrary.com]

The parameters required to fully characterize the Knoxville treatment machine at all energies were implemented in matRad: namely, *α*,* γ*,* V* and the integrated dose at a distance *z* along the beam path. Figure 7 compares a matRad‐calculated beam with a fully commissioned Raysearch planning system.

## 4 Discussion

The use of stable distributions provides a way of calculating the dose in a medium in a scanned pencil beam proton therapy machine that lends itself to implementation on GPU‐type architectures. The calculation in the Fourier space can be done fast and libraries that perform fast Fourier transforms on such processors, are readily available. Alternatively, it is possible to directly estimate the integral provided by the inverse transform yielding:

(18) $$f\left(x\right)=\int_{0}^{\infty }{\text{exp}(-|\gamma t|}^{\alpha })\text{cos}\left(xt\right)dt$$

This can be numerically evaluated using a Gaussian Quadrature method, which is computationally faster than a fast Fourier transform^22^. Although Monte Carlo simulation‐type dose calculators are becoming increasingly available, the use of an analytical alternative is interesting when many recalculations of treatment plans are needed. To note, this is important in cases where either fast calculations are needed (adaptive treatment) or multiple scenarios need to be evaluated (4D‐planning and/or 4D‐robust‐optimization).

Providing a parameterization of this type reduces the number of parameters to a more manageable level. Moreover, the parameters are well behaved as a function of depth and energy, allowing a better insight in the physics of proton therapy planning using scanned pencil beams. It becomes clear, for instance, that the scattering properties of combined scanned beams are different depending on the depth of the treated volume. This is described by the parameter *α* and its behavior at different depths and nominal energies. Therefore, different dose characteristics, depending on the combination of pencil beam energies, can be expected. It also provides a method to describe issues like changes in medium in terms of the used parameters. In a forthcoming study, we have already established that not all parameters behave in the same manner as a function of depth combined with changes in material (data not shown). An added advantage of a minimal number of parameters is the reduction of co‐variance of the parameters, leading to a unique description of the evolution of the dose distribution with depth. A good example for this is the robustness of the parameters with respect to environment variables like detector size. This was shown not to be the case with other approaches like, for instance, the multiple Gaussian approach.

In this current study, we considered the pencil beam to be radially symmetric. In practice it is possible that this is not the case depending on the geometric properties of the machine used to generate the pencil beams. For instance, many proton therapy facilities will use spatially sequential magnets to bend the beams in the directions perpendicular to the beam axis in two perpendicular directions to each other. This results in an ellipsoid spot size due to a different virtual source position associated with each scanning direction. It is also observed that when using gantries, the ellipsoidal nature of the beam spot can change. The implication is that we have to find a way to combine different stable distributions. In the case of the normal distribution this is well understood, that is, combining the variations depending on the mixing angle. Combining generalized stable distributions is less straightforward, but still fairly trivial in the case where the *α* parameter is constant. Investigating Eq. (5) shows that the combination of two distributions with the same *α* and scale parameters *γ*_1_, and *γ*_2_ yields a new stable distribution with scale parameter *γ*:

(19) $$\gamma ={\left(\gamma_{1}^{\alpha }+\gamma_{2}^{\alpha }\right)}^{\frac{1}{\alpha }}$$

Fortunately, we have seen that the parameter *α* depends only on the amount of material that has been passed. As a result the value of *α* is the same in every direction of the plane. Combining stable distributions with different *α* is not straightforward because as far as we know the resulting distribution is not stable and is still an area of mathematical research.

We have also limited this study to that of symmetric zero‐centered pencil beams. While the zero‐centering is easily resolved by a well chosen coordinate transformation, the asymmetry of a pencil beam is not resolvable in an easy way. Indeed, in some cases the treatment beams are not symmetric, specifically if collimation is used and pencil beams near the collimator jaws need to be considered. In that case the parameter *β* is not zero and the full expression as outlined in Eq. (4) needs to be evaluated. This is subject of further research by our group.

In theory, the methodology we have shown here could be extended to other charged particles and photons, but this is yet to be substantiated.

## 5 Conclusion

We have demonstrated that stable distributions are suited to describe charged particle pencil beams in a medium. For all energies we found that the agreement expressed as a *χ* ^2^/NDF was of the order of 1, indicating excellent prediction. Only for the highest energy (226 MeV) higher values of 10 were seen. This could be resolved by adding another stable distribution with *α* = 2 (i.e., a normal distribution). We have shown how this parameterization of the pencil beam allows dose distributions from intermediate energies to be interpolated through intermediate morphing and that this yields very good agreement. The parameters required to fully characterize the Knoxville beam at all energies were also implemented in matRad and the obtained dose depositions were well comparable with the results from the Monte Carlo and TPS. Finally, we showed that the parameterization is robust in terms of detector size, a property not available in the more traditional approaches.

## Contributions

Frank Van den Heuvel: Concept, fitting the stable distributions, and editing the article; Francesca Fiorini: Monte Carlo simulations and cowriting the article; Niek Schreuder: Measurements to verify pencil beam Monte Carlo calculations, and cowriting the article; Ben George: Implementation of the algorithm in matRad, incorporating stable distribution calculations in C, and cowriting the article.

## Supporting information


**Data S1.** Stable distributions in protons.

## References

[1] Taylor PA , Kry SF , Followill DS. Pencil beam algorithms are unsuitable for proton dose calculations in lung. Int J Radiat Oncol Biol Phys. 2017;99:750–756. 28843371 10.1016/j.ijrobp.2017.06.003 PMC5729062

[2] Fiorini F , Hackett S , Van den Heuvel F. EP‐1458: proton breast treatments: eclipse vs Monte Carlo Fluka dose comparison study. Radiother Oncol. 2015;115:S790.

[3] Gottschalk B , Cascio EW , Daartz J , Wagner MS . Nuclear halo of a 177 MeV proton beam in water: theory, measurement and parameterization. *ArXiv e‐prints*; September 2014.

[4] Gottschalk B , Cascio EW , Daartz J , and Wagner MS. On the nuclear halo of a proton pencil beam stopping in water. Phys Med Biol. 2015;60:5627. 26146956 10.1088/0031-9155/60/14/5627

[5] Pedroni E , Scheib S , Bhringer T , et al. Experimental characterization and physical modelling of the dose distribution of scanned proton pencil beams. Phys Med Biol. 2005;50:541. 15773729 10.1088/0031-9155/50/3/011

[6] da Silva J , Ansorge R , Jena R. Fast pencil beam dose calculation for proton therapy using a double‐gaussian beam model. Front Oncol. 2015;5:281. 26734567 10.3389/fonc.2015.00281 PMC4683172

[7] Raystation 4.7 Reference Manual . Technical report, RaySearch Laboratories AB; 2014.

[8] Bellinzona VE , Ciocca M , Embriaco A , et al. On the parametrization of lateral dose profiles in proton radiation therapy. Phys Med: Eur J Med Phys. 2015;31:484–492. 10.1016/j.ejmp.2015.05.004 26032003

[9] Nolan JP . Maximum likelihood estimation and diagnostics for stable distributions In: Barndorff‐Nielsen OE, Mikosch T, Resnick SI, eds. Lévy Processes: Theory and Applications. Boston, MA: Birkhäuser Boston; 2001:379–400.

[10] Uchaikin VV , Zolotarev V . Elementary introduction to the theory of stable laws In: Kolorev VYu, Zolotarev VM, eds. Chance and Stability: Stable Distributions and their Applications. Berlin, Boston: De Gruyter; 1999:35–64.

[11] Humphries NE , Queiroz N , Dyer JRM , et al. Environmental context explains Lévy and Brownian movement patterns of marine predators. Nature. 2010;465:1066–1069. 20531470 10.1038/nature09116

[12] Fassò A , Ferrari A , Ranft J , Sala PR . FLUKA: a multi‐particle transport code, CERN‐2005‐10. *INFN/TC_05/11, SLAC‐R‐773*; 2005.

[13] Battistoni G , Muraro S , Sala PR , et al. The FLUKA code: description and benchmarking. Proceedings of the Hadronic Shower Simulation Workshop 2006, Fermilab 6–8 September 2006, M. Albrow, R. Raja eds., AIP Conference Proceeding; 2007;896:31–49.

[14] Fiorini F , Schreuder N , Van den Heuvel F. Technical note: defining cyclotron‐based clinical scanning proton machines in a FLUKA Monte Carlo system. Med Phys. 2018;45:963–970. 29178429 10.1002/mp.12701 PMC6571526

[15] Mittnik S , Rachev ST , Doganoglu T , Chenyao D. Maximum likelihood estimation of stable paretian models. Math Comput Modell. 1999;29:275–293.

[16] Julián‐Moreno G , Lópezd˜e Vergara JE , González I , de Pedro L , Royuela‐del Val J , Simmross‐Wattenberg F. Fast parallel *α*–stable distribution function evaluation and parameter estimation using OpenCL in GPGPUs. Stat Comput. 2016;5:1–18.

[17] Andrae R , Schulze‐Hartung T , Melchior P . Dos and don’ts of reduced chi‐squared. *ArXiv e‐prints*; December 2010.

[18] Press WH , Teukolsky SA , Vetterling WT , Flannery BP (eds). Modeling of data In Numerical Recipes: The Art of Scientific Computing. Cambridge: Cambridge University Press; 1993.

[19] Marquardt DW. An algorithm for least‐squares estimation of nonlinear parameters. J Soc Ind Appl Math. 1963;11:431–441.

[20] Cisternas E , Mairani A , Ziegenhein P , Jäkel O , Bangert M . matrad‐a multi‐modality open source 3d treatment planning toolkit In: Jaffray D, ed. World Congress on Medical Physics and Biomedical Engineering, June 7‐12, 2015. Toronto, Canada: Springer; 2015:1608–1611.

[21] Wieser H‐P , Cisternas E , Wahl N , et al. Development of the open‐source dose calculation and optimization toolkit matrad. Med Phys. 2017;44:2556–2568. 28370020 10.1002/mp.12251

[22] Belov IA. On the computation of the probability density function of *α*‐stable distributions. Math Modell Anal. 2005;2:333–341.

