# Supplementary material for: Using stable distributions to characterize proton pencil beams
Source: Med Phys. 2018 Apr 15;45(5):2278–88. doi: 10.1002/mp.12876 (PMC6571675; doi:10.1002/mp.12876)
Supplement: Supplementary file 1 — Data S1. Stable distributions in protons. [file MP-45-2278-s001.pdf]

Supporting Material I

The data measured at CNAO was kindly provided by Andrea Fontana from the INFN at Pavia, Italy. We performed fits for all energies available. In contrast with the simulation data the measurement data was sometimes slightly shifted from the central axis. Rather than manually shift the data we incorporated it as an extra parameter δ in the stable distribution fit.

Energy (MeV)	Depth(mm)	α	δ	γ
118	25	1.938	-4.4	3.99
118	50	1.927	-4.4	4.13
118	80	1.921	-4.3	4.39
118	90	1.923	-4.3	4.48
118	98	1.933	-4.3	4.61
131	25	1.932	0.4	3.79
131	50	1.908	0.4	3.91
131	100	1.902	0.5	4.30
131	115	1.896	0.4	4.50
131	118	1.905	0.4	4.55
131	120	1.936	0.3	4.59
143	25	1.921	0.6	3.57
143	50	1.890	0.6	3.65
143	100	1.880	0.6	4.01
143	132	1.885	0.6	4.41
143	138	1.892	0.6	4.51
143	140	1.924	0.6	4.55

Energy (MeV)	Depth(mm)	α	δ	γ
154	25	1.912	1.0	3.37
154	50	1.876	1.0	3.47
154	100	1.862	1.0	3.79
154	152	1.872	0.8	4.42
154	158	1.883	0.8	4.55
154	160	1.913	0.8	4.59
164	25	1.900	0.9	3.16
164	60	1.854	0.8	3.29
164	130	1.845	0.8	3.82
164	172	1.868	-3.6	4.48
164	178	1.887	-3.6	4.65
164	180	1.930	-3.6	4.63
174	25	1.910	-3.4	3.04
174	70	1.843	-3.6	3.19
174	150	1.855	-3.6	3.90
174	190	1.873	-3.4	4.55
174	198	1.891	-3.3	4.73
174	200	1.921	-3.5	4.73

Table 1: All measured data fit using Nolan's spline method

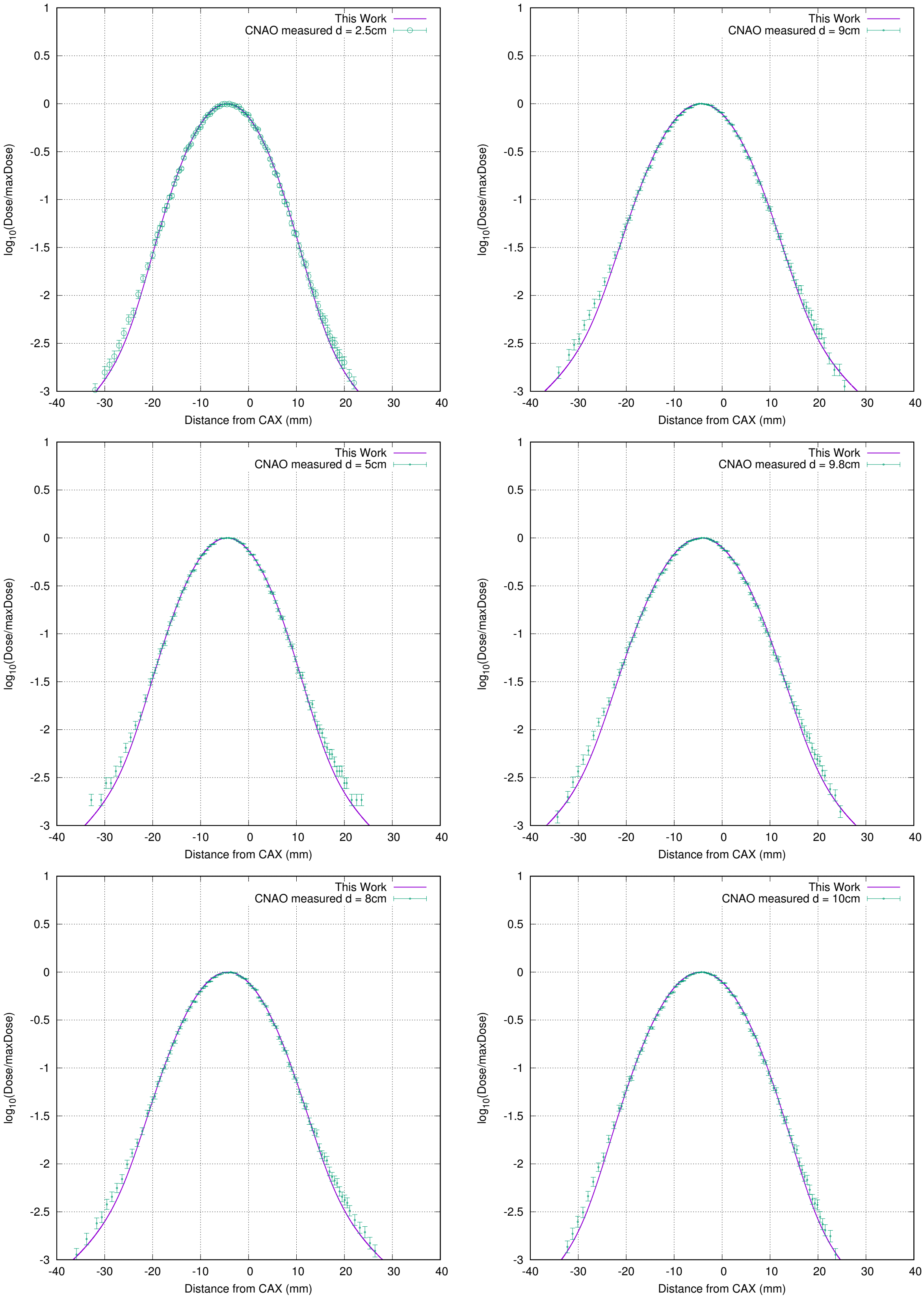

Figure 1: 118MeV

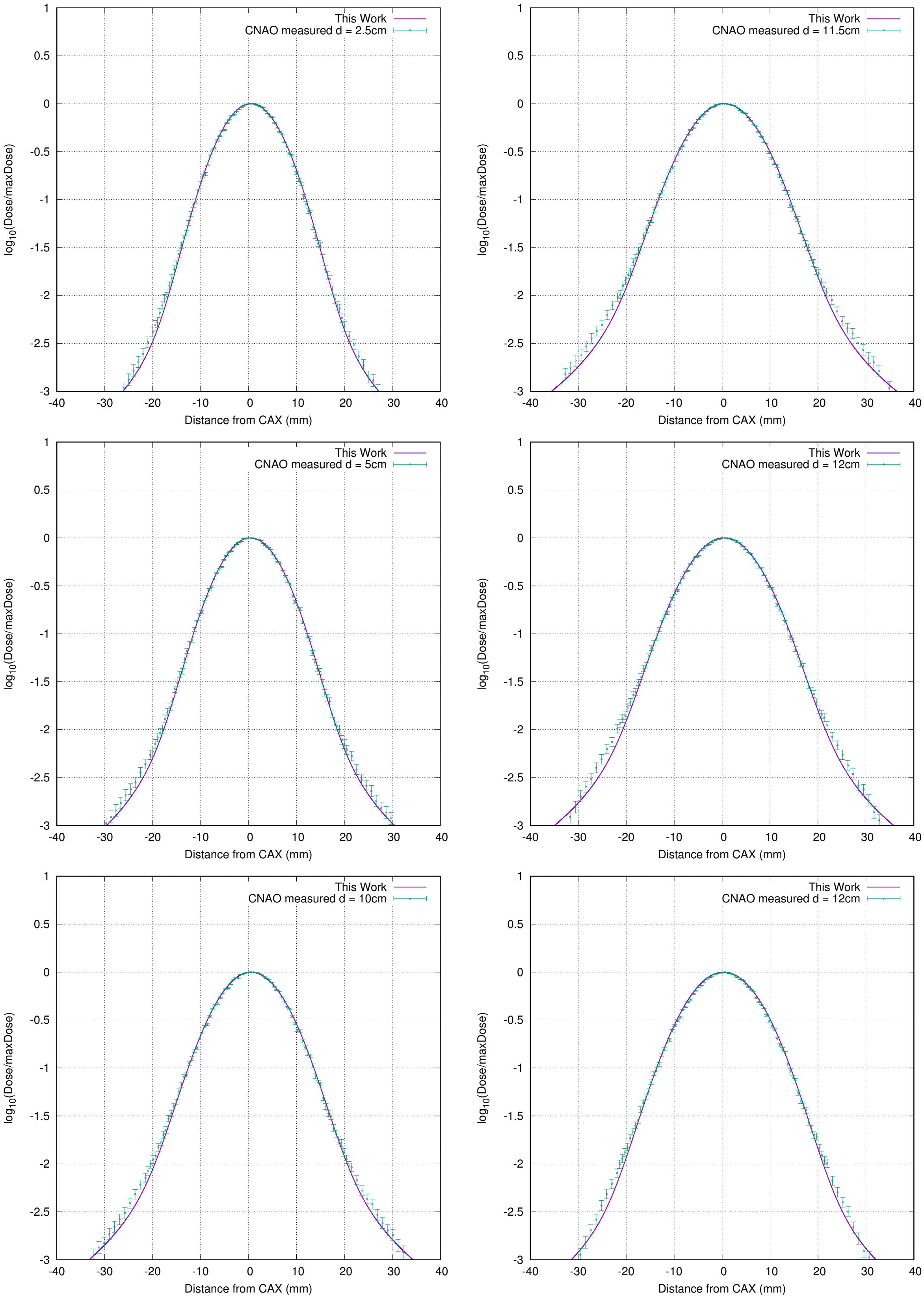

Figure 2: 131MeV

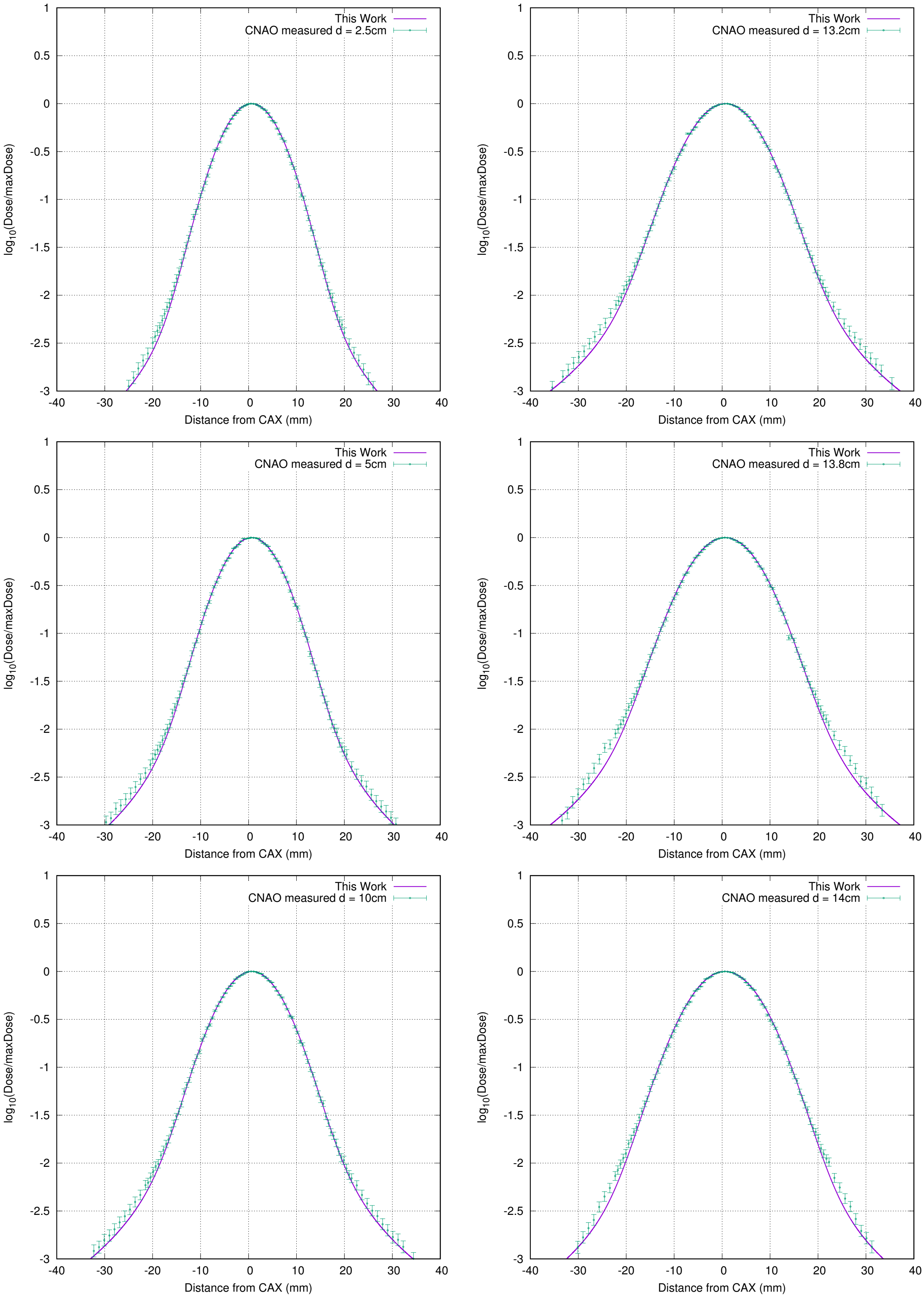

Figure 3: 143MeV

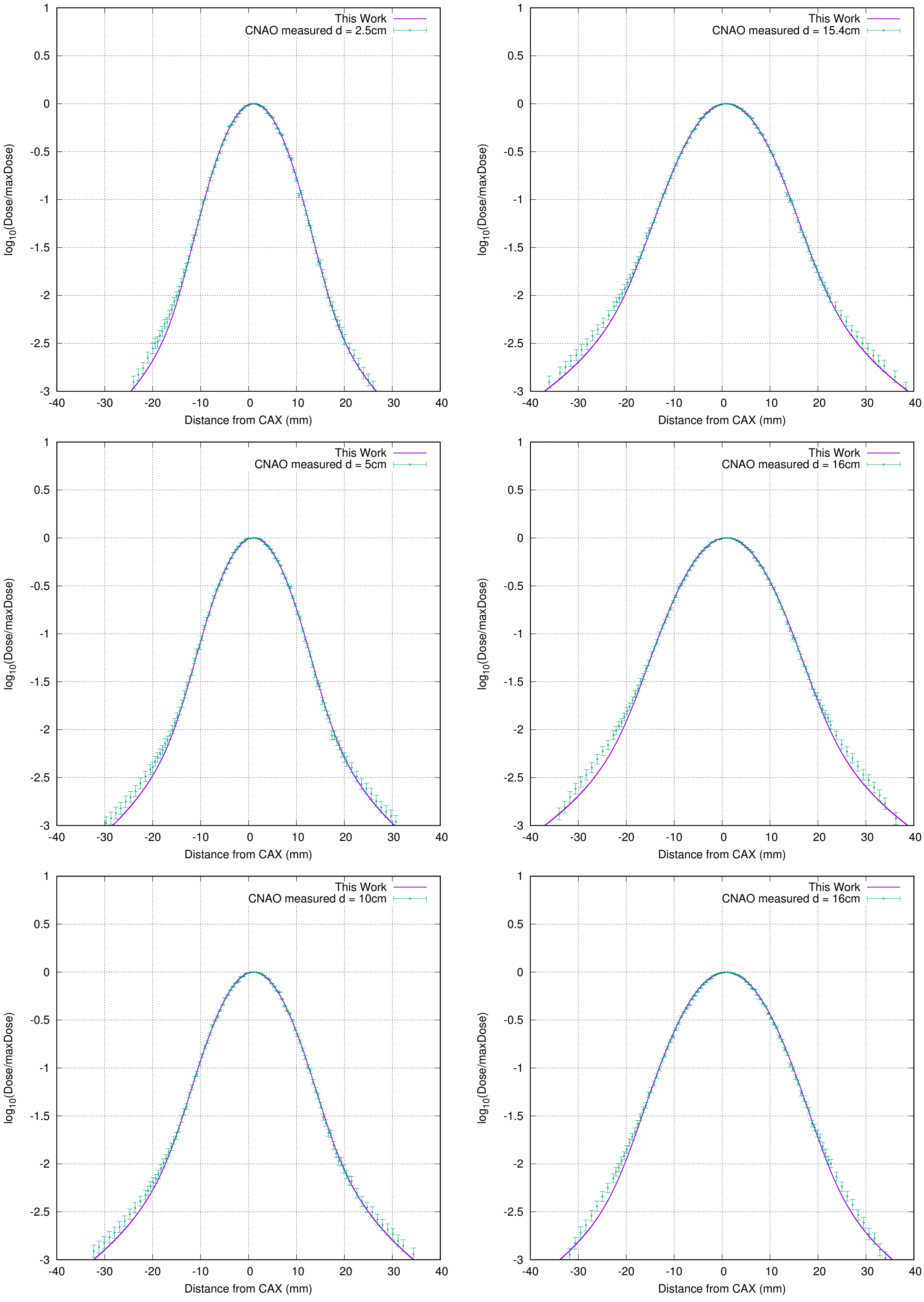

Figure 4: 154MeV

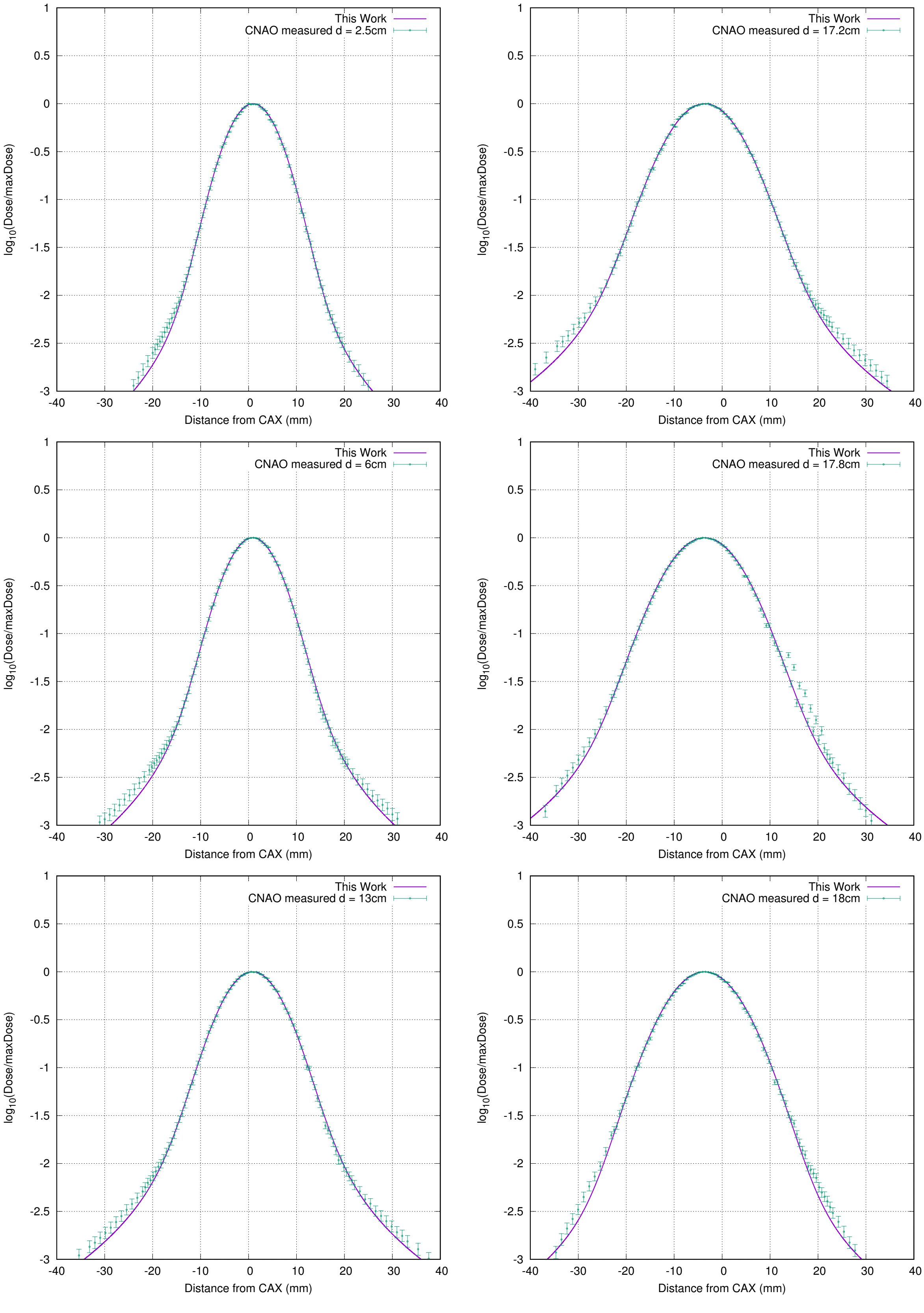

Figure 5. 164MeV

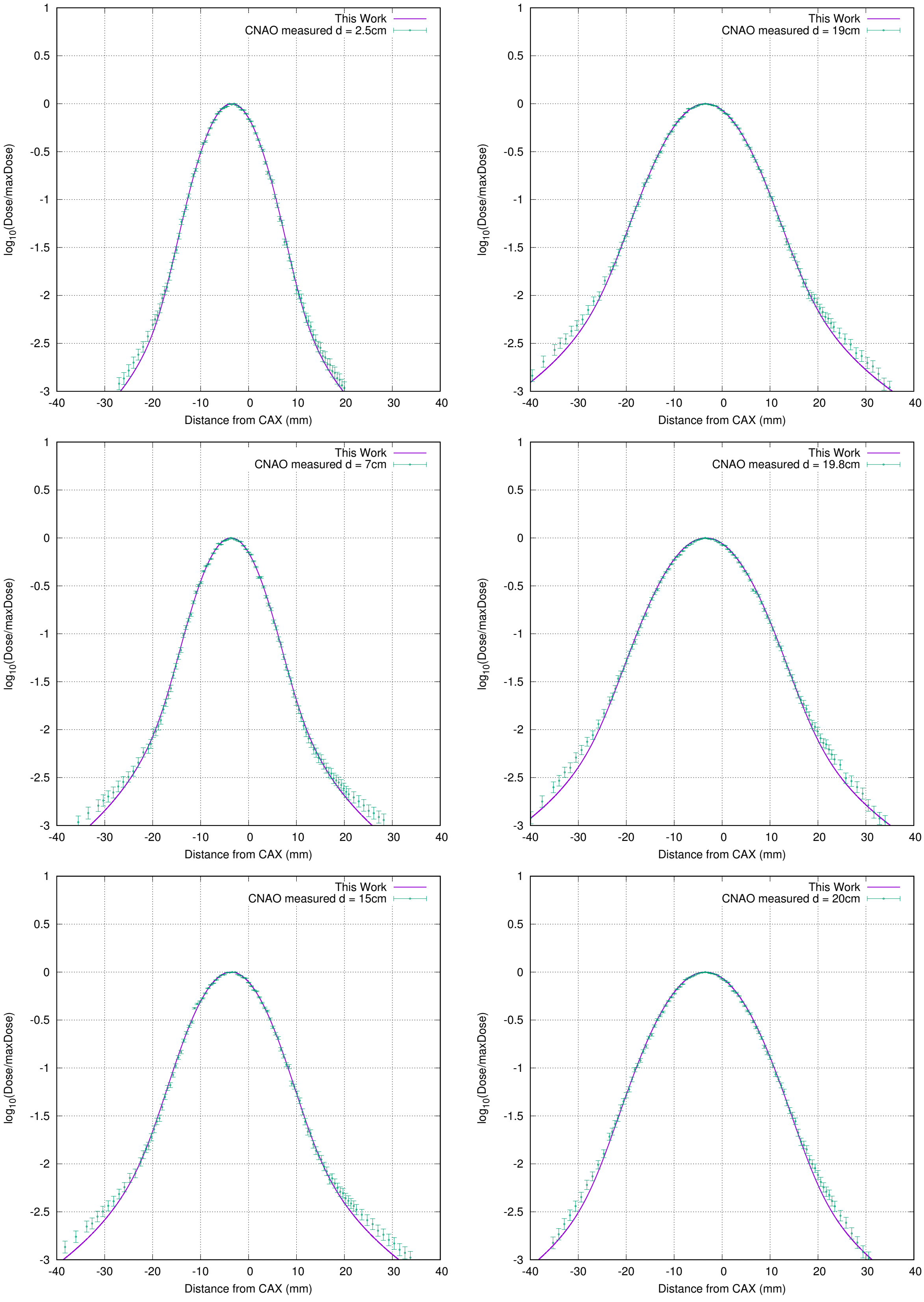

Figure 6: 174MeV

Supporting Material II

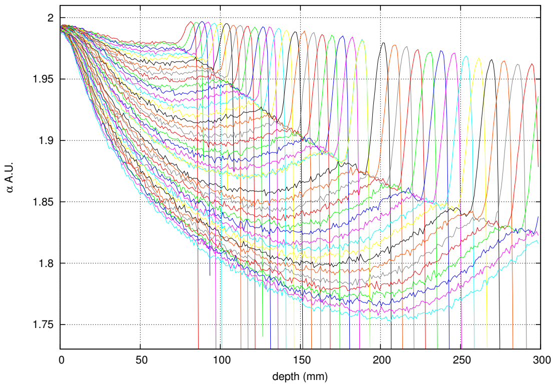

(a) $\alpha(z)$

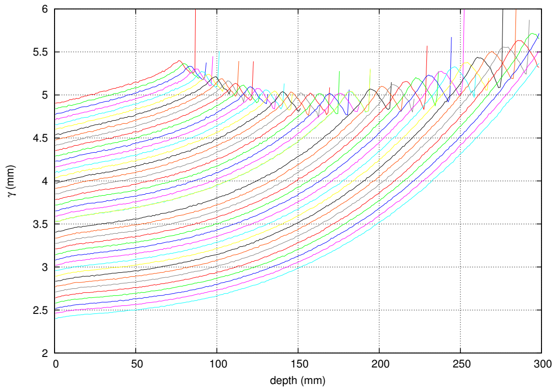

(b) $\gamma(z)$

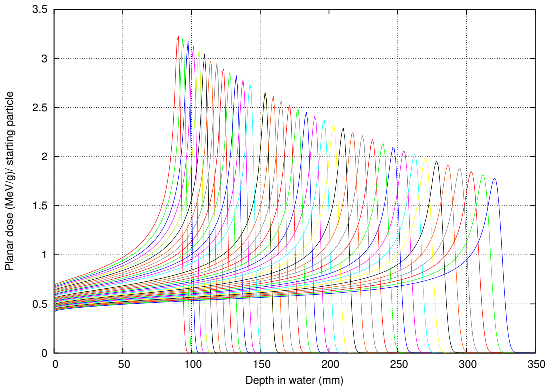

(c) $I_D(z)$

Figure 7: From top to bottom: The evolution of α, γ, and integrated dose, note that the graph for each energy has the same general shape

Supporting Material III

In this appendix we outline the notion of stable distributions, provide some definitions and show that the characteristic representation parameterizing the quantities (α and γ) indeed represents all stable distributions. The text is extensively based on the treatise by Uchaikin and Zolotarev and is provided as a synthesis and guideline rather than an original scientific contribution, the original work is much more extensive and dense [1].

Defining stable distributions

We start out by quoting the law of large numbers which states that the difference between the estimated mean of a sample from a random variable tends to the mean of the distribution when enough samples are taken. It is best known in the form as proposed by Bernouilli in the 18th century:

Theorem 1 (law of large numbers — Jacob Bernoulli)**.** *Let X_1, X_2,$\ldots X_n$ be independent, identically distributed random variables with mean $\mu_n = \frac{1}{n}\sum_{i=1}^{n} X_i$ then:*

$$P\{|\mu_n - p| \geq \epsilon\} \to 0, n \to \infty \tag{1}$$

Since

$$\epsilon_n = \mu_n - p \xrightarrow{P} \infty \tag{2}$$

i.e. it converges to zero in probability as $n \to \infty$, which provides the reformulation of Bernoulli's law of large numbers:

$$\mu_n = p + \epsilon_n, n \geq 1. \tag{3}$$

A more sophisticated approach considers the random variables as functions on an interval [0,1] where ω is an instantiation of the experiment yielding 0 or 1. In that case the strong law of large numbers can be replaced by a weaker version:

$$\mu_n = \sum_{i=1}^{n} X_i(\omega) \tag{4}$$

which then becomes a degenerate function if infinite samples are taken, but more importantly, before reaching the degenerate condition the sum tends to the normal distribution, which is the classical form of the the central limit theorem.

Theorem 2 (central limit theorem — Moivre–Laplace)**.** *Let X_1, X_2,$\ldots$ be independent, identically distributed random variables with mean μ and variance $\sigma^2 < \infty$. Then as $n \to \infty$,*

$$P\left\{\frac{\sum_{i=1}^{n} X_i - n\mu}{\sigma\sqrt{n}} < x\right\} \Rightarrow \phi(x) = \int_{-\infty}^{x} p^G(x)dx \tag{5}$$

Where

$$p^G(x) = \frac{1}{\sqrt{2\pi}} e^{-x^2/2}$$

We also provide the notion of *equivalent* distributions X and Y:

$$X \stackrel{d}{=} Y \iff p_X(x) = p_Y(x) \tag{6}$$

As well as the notion of similar distributions which provides the possibility of introducing a linear transformation of the given distribution.

$$X \stackrel{s}{=} Y \iff Y \stackrel{d}{=} a + bX \tag{7}$$

Using expression 6 and 7 it is clear that for similar distributions X and Y on an infinitesimal interval dx:

$$p_Y(x)dx = p_{a+bX}(x)dx = p_X\left(\frac{x-a}{b}\right)\frac{dx}{b} \tag{8}$$

Therefore, the same applies to the distribution functions (cumulative of the density function):

$$F_{a+bX}(x) = F_X\left(\frac{x-a}{b}\right) \tag{9}$$

For example the normal distribution $p^G(x;0,1) = \frac{1}{\sqrt{2\pi}}\exp(-x^2/2)$ provides the following expression:

$$p^G(x;a,\sigma) = \frac{1}{\sigma}p^G(\frac{x-a}{\sigma}) \tag{10}$$

An interesting property arises when, instead of looking at the variables themselves, we now investigate how sums of these variables (summands) behave. If we have two normal distributions Y_1 and Y_2 with variances σ_1 and σ_2 then it is easy to see that, using expression 10, we get:

$$\sigma_1 Y_1^G + \sigma_2 Y_2^G = \sqrt{\sigma_1^2+\sigma_2^2} \times Y^G \tag{11}$$

By setting $\sigma_1 = \sigma_2 = 1$ and with an arbitrary summands, n, we obtain a well known result:

$$\sum_{i=1}^{n} Y_i^G \stackrel{d}{=} \sqrt{n} \times Y^G \tag{12}$$

Or more interestingly expressed as:

$$\sum_{i=1}^{n} Y_i^G \stackrel{s}{=} Y^G, a = 0, b = \sqrt{n} \tag{13}$$

It is the generalization of this property that leads to the notion of stable distributions by allowing arbitrary values of a and b.

Definition 1. *A random variable Y is stable if and only if for any arbitrary constants b' and b'' there exist constants a and b such that:*

$$b'Y_1 + b''Y_2 = a + bY \tag{14}$$

Which then leads to the stable law in the same form as the central limit theorem, as shown by the proof below.

Theorem 3 (Lévy)**.** *Let X_1, X_2,... be independent, identically distributed random variables, and let there exist constants $b_n > 0$ and a_n such that*

$$P\left\{\frac{\sum_{i=1}^{n} X_i - a_n}{b_n} < x\right\} \Rightarrow G(x), n \to \infty \tag{15}$$

for some function $G(x)$ which is not degenerate[1]*. Then $G(x)$ follows the stable law.*

P represents the given probability at the value x. While $G(x)$ is the cumulative probability density function of the distribution.

α, β representation

In this section, we show how we move from the expression in 15 to the parameterization that we have been using denoting the type of stable distribution based on the single parameter α. Before we move on, we narrow the definition of stable distributions to that of *strictly* stable distribution by setting $a_n = 0$, thus:

$$S_n = \sum_{i=1}^{n} Y_i \stackrel{d}{=} b_n Y. \tag{16}$$

[1]By not degenerate we imply that: $G(x)$ is not a step function

Defining S_n the distribution of the sum. By calculating the variance of the distribution 16 we obtain:

$$n \times \mathrm{var}(Y) = b_n^2 \times \mathrm{var}(Y) \tag{17}$$

If $\mathrm{var}(Y) \neq 0$ and $\mathrm{var}(Y) < \infty$ then there is only one possibility

$$b_n \equiv b_n^G = n^{1/2} \tag{18}$$

Which reverts to the result obtained for the normal distribution.

With the notion of summands, we can now use them to further extend the properties to the general case of summing strictly stable random variables.

$$\begin{aligned} Y_1 + Y_2 &\stackrel{d}{=} b_2 X \\ Y_1 + Y_2 + Y_3 &\stackrel{d}{=} b_3 X \\ Y_1 + Y_2 + Y_3 + Y_4 &\stackrel{d}{=} b_4 X \end{aligned} \tag{19}$$

We now choose to limit ourselves to summands with 2^k terms

$$\begin{aligned} Y_1 + Y_2 &\stackrel{d}{=} b_2 X \\ Y_1 + Y_2 + Y_3 + Y_4 &\stackrel{d}{=} b_4 X \\ Y_1 + Y_2 + Y_3 + Y_4 + Y_5 + Y_6 + Y_7 + Y_8 &\stackrel{d}{=} b_8 X \\ &\dots \\ Y_1 + Y_2 + \ldots + Y_{2^{k-1}} + Y_{2^k} &\stackrel{d}{=} b_{2^k} X \\ &\dots \end{aligned} \tag{20}$$

Keeping in mind that $X_1 + X_2 \stackrel{d}{=} X_3 + X_4$, which we can generalize to any pair, we can rewrite the expression in (20) to read:

$$\begin{aligned} Y_1 + Y_2 &\stackrel{d}{=} b_2 X \\ Y_1 + Y_2 + Y_3 + Y_4 &\stackrel{d}{=} {b_2}^2 X \\ Y_1 + Y_2 + Y_3 + Y_4 + Y_5 + Y_6 + Y_7 + Y_8 &\stackrel{d}{=} {b_2}^3 X \\ &\dots \\ Y_1 + Y_2 + \ldots + Y_{2^{k-1}} + Y_{2^k} &\stackrel{d}{=} {b_2}^k X \\ &\dots \end{aligned} \tag{21}$$

Or in much shorter notation

$$S_{2^k} = b_{2^k} Y = b_2^k Y \tag{22}$$

and using the expression in defining strictly stable random variables16: $S_n = b_n Y$, we obtain:

$$b_n = b_2^k = b_2^{(\ln n)/\ln 2} \tag{23}$$

transforming into

$$\ln b_n = [(\ln n)/\ln 2] \ln b_2 = \ln n^{(\ln b_2)/\ln 2} \tag{24}$$

or

$$b_n = n^{(\ln b_2)/\ln 2} = n^{1/\alpha_2} \tag{25}$$

We can now repeat this process with 3^k, 4^k and using induction m^k, yielding α_3, α_4, and α_m, bunching respectively 3, 4 and m summands. In general the following expression is valid:

$$b_n = n^{1/\alpha_m},\ \alpha_m = (\ln m)/\ln b_m \tag{26}$$

For arbitrary values of m.

If we now set $m = 4$ we get

$$\alpha_4 = (\ln 4)/\ln b_4$$

On the other hand, selecting $k = 2$ in expression (25), yields

$$\ln b_4 = 1/\alpha_2 \ln 4$$

From these last two formula we conclude that $\alpha_2 = \alpha_4$. By induction we see that there is a single α for all these stable distributions, and that the scaling factors b_n follow the following law:

$$b_n = n^{1/\alpha} \tag{27}$$

Characteristic function of symmetrical zero–centered stable distribution

The characteristic function, c.f., of a distribution can be defined as the Fourier transform of the probability density function, p.d.f., of that distribution. Let $p_X(x)$ be a p.d.f. of a set of random variables X, then the c.f., $f_p(k)$, is defined as it's expectation value, $e^{\mathrm{i}kX}$:

$$f_X(k) = \int_{-\infty}^{\infty} p_X(x)e^{\mathrm{i}k}dx \tag{28}$$

From this definition some properties follow immediately:

1. $f_X(0) = 1$
2. $f_{a+bX} = e^{\mathrm{i}ka} f_X(bk)$
3. $f_X^*(k) = f_X(-k) = f_{-X}(k)$, where $*$ denotes the complex conjugate.
4. If X is symmetric about zero,
$$X \stackrel{d}{=} -X$$
5. If $\mathbf{E}|X|^n,\ n \geq 1$ then the continuous nth derivative of the c.f. exists and:
$$f^{(n)}(0) = \mathrm{i}^n \mathbf{E}X^n$$
6. if S_n is the sum of independent random variables X_1, X_1, ..., X_n, then:
$$f_{S_n}(k) = f_{X_1}(k) \ldots f_{X_n}(k)$$
7. any $f_X(k)$ is a uniformly continuous function.

In order to progress further, we invoke the inversion theorem to be able to find the distribution function given the c.f.

Theorem 4. *(Inversion theorem) Any distribution function $F(x)$ is uniquely defined by its c.f. $f(k)$. If a and b are some continuity points if $F(x)$, then the inversion formula states that*

$$F(b) - F(a) = \lim_{c\to\infty} \frac{1}{2\pi} \int_{-c}^{c} \frac{e^{-\mathrm{i}kb} - e^{\mathrm{i}ka}}{ik} f(k)dk \tag{29}$$

The principle advantage of using the c.f. is that the c.f. of a sum of independent random variables is equal to the product of the c.f.s of the summands:

$$f_{X_1+X_2}(k) = f_{X_1}(k) f_{X_2}(k), \tag{30}$$

If we take the logarithm (obtaining the second characteristic: $\psi_X(k) = \ln f_X(k)$), we find that:

$$\psi_{X_1+X_2}(k) = \psi_{X_1}(k) + \psi_{X_2}(k) \tag{31}$$

This is important as it allows us to assess the summation of a large number of independent random variables without evaluating multiple integrals. For this reason we cite the continuity theorem:

Theorem 5. *(continuity theorem) Let $f_n(k), n = 1, 2, \ldots$ be a sequence of c.f.s and let $F_n(x)$ be a sequence of corresponding distribution functions. If $f_n(k) \to f(k)$ as $n \to \infty$, for all k and $f(k)$ is continuous at $k = 0$, then $f(k)$ is the c.f. of a cumulative distribution function $F(x)$, and the sequence $F_n(x)$ weakly converges to $F(x)$, $F_n \Rightarrow F$. The inverse is also true: if $F_n \Rightarrow F$ and F is a distribution function, then $f_n(k) \to f(k)$, where $f(k)$ is the c.f. of the distribution function F.*

To gain some insight in how to perform this, we can look at two well known stable distributions to find a way forward. The distributions under consideration are the normal distribution and the Cauchy distribution. The calculation of the characteristic function for these distributions is well known and they also form part of the stable distribution, in the form $q(x; \alpha, \beta)$ representing the stable distribution density:

Normal distribution $q(x; 2, 0) = \frac{1}{2\sqrt{\pi}} e^{-x^2/4}$

Cauchy distribution $q(x; 1, 0) = \frac{1}{\pi} \frac{1}{1+x^2}$

The characteristic function, $g(k; \alpha, \beta)$ in this notation, can then be calculated in a straightforward manner and can be found in many textbooks:

Normal distribution $g(k; 2, 0) = e^{-k^2}$

Cauchy distribution $g(k; 1, 0) = e^{-|k|}$

Note that the traditional form of the density function for the normal distribution is slightly different:

$$p^G(x) = \frac{1}{\sqrt{2\pi}} e^{-x^2/2} \tag{32}$$

This observation allows us to generalize for any symmetric distributions. Let Y_i be such a distribution with an arbitrary parameter α. We remind that

$$\sum_{i=1}^{n} Y_i \stackrel{d}{=} b_n Y, \quad b_n = n^{1/\alpha} \tag{33}$$

Keeping in mind the property as elucidated in equation 30

$$f_Y^n(k) = f_Y(n^{(1/\alpha)} k) \tag{34}$$

using the expression for the second characteristic

$$n\psi_Y(k) = \psi_Y(n^{(1/\alpha)} k) \tag{35}$$

The results obtained earlier when investigating the normal- and the Cauchy distribution lead us to propose a solution of the form

$$\psi_Y(k) = -ck^{\mu}, \quad k > 0 \tag{36}$$

This satisfies the previous expression with $\mu = \alpha$ and an arbitrary complex–valued c, which we choose to be

$$c = \lambda[1 - \mathrm{i}c_1], \quad \lambda, c_1, \text{ real numbers} \tag{37}$$

To find $\psi_Y(k)$ we use property (3) of the chacaracteristic function, the link between conjugate characteristic function and negative estimates:

$$\psi_Y(k) = -\lambda[|k|^\alpha - \mathrm{i}k\omega(k)], \quad -\infty < k < \infty, 0 < \alpha \leq 2 \tag{38}$$

with

$$\omega(k) = c_1|k|^{\alpha-1} \tag{39}$$

Which is a trick to rewrite the equation as $k \times k^{\alpha-1} = k^\alpha$, in such a way that we are explicitly splitting the expression in a real and an imaginary part by a good choice of the constant c. We also have not specified what form the function $\omega(k)$ takes, we do know that it will depend on the parameter α as well as provide a measure for the asymmetry of the distribution, should that be present. Later we will attribute that to a parameter β. The constant λ is an arbitrary real number and can serve as a scaling factor, which can be renormalised to 1, without loss of generality. This implies that the full expression of a stable distributions characteristic function is of the form:

$$g(k; \alpha, \beta) = \exp(-|k|^\alpha + \mathrm{i}k\omega(k; \alpha, \beta)) \tag{40}$$

Taking into account that the characteristic function of a symmetric stable function is real–valued due to property (3) of the characteristic functions:

$$\omega(k; \alpha, \beta) = 0 \tag{41}$$

and the characteristic function for any stable function becomes:

$$\boxed{g(k; \alpha, 0, \gamma) = \exp(-|\gamma k|^\alpha)} \tag{42}$$

With γ as a scaling factor.

[1] Vladimir V. Uchaikin and Vladimir Zolotarev. *Chance and Stability: Stable Distributions and their Applications*, chapter Elementary introduction to the theory of stable laws, pages 35–64. De Gruyter, Berlin, Boston, 1999.
